# Supplementary material for: Patterns of Intron Gain and Loss in Fungi
Source: PLoS Biol. 2004 Nov 30;2(12):e422. doi: 10.1371/journal.pbio.0020422 (PMC532390; doi:10.1371/journal.pbio.0020422)
Supplement: Table S1 — Also available at http://genes.mit.edu/NielsenEtAl/. (4.3 MB ZIP). [file pbio.0020422.st001.zip › NielsenEtAl/html/170.html]

AN6273.1.NCU07787.1.MG05344.1.FG11205.1


```
 CLUSTAL W (1.82) Multiple Sequence Alignments - Introns Inserted


Sequence 1: NCU07787.1	138 aa
Sequence 2: MG05344.1	137 aa
Sequence 3: FG11205.1	140 aa
Sequence 4: AN6273.1	158 aa
Alignment Length: 159 aa
Number Identitical Residues: 48 aa
Alignment Score (without introns) 2820


MG05344.1 	MQFSNILSIFTLAAAAQ------------AVSV~SYDTGYDDGSRSLTAVSCSDGANGLI
NCU07787.1	MQLSKILSLFTLATAAS------------ATTV1SYDTGYDDPNRSLTVVSCSDGSNGLI
FG11205.1 	MQLTNLFCLASVLTSVS------------AITV1SYDPGYGEAGRAMTAVSCSDGTNGLI
AN6273.1  	MKLATLLPAFAAAALAAPSNPLVARQTSGTSTL~SYDTKYDVADTSLTTVACSDGDYGLI
          	*:::.::   :  : . .:..  : .::.: :: ***. *.  . ::*.*:****  ***

MG05344.1 	TKYGWQTQGQIRNFPYIGGVDAVGGWNSPS0CGTCWQLTYN---GKS--INVLAIDHA-S
NCU07787.1	TKYHWNFQNQVKNFPYIGGVEAVAGWNSPN~CGTCWSVTYN---GKT--INILAIDHAGA
FG11205.1 	TRYGWKTQGQIPKFPYIGGAQAIAGWNSPS~CGTCWKLTYK---GKS--INVLAIDHTAA
AN6273.1  	T-EGYTTFGSLPTFARIGGAPTIPGWNSPN~CGSCYQITYTSATGQTNSIIMTAIDTAPG
          	*   :   ..: .*. ***. :: *****. **:*:.:**.:::*::.:* : *** : .

MG05344.1 	GFNIGLAAMNDLTNGQAGSLGRIEAQSQQVGLNACGL----
NCU07787.1	GVNLSKKAMNELTGGNAEQFGRVDAQVQQVALSACGL----
FG11205.1 	GFNISPAAMNALTNNQAVQLGRVDATATQVAVSNCGLKK--
AN6273.1  	GFNVGQQAMDLLTGGRAAELGRVDITWTQVGREQCGLTPRP
          	*.*:.  **: **...* .:**::    **. . ***.  .
```
